# Glucose variability as a key mediator in the relationship between pre-pregnancy overweight/obesity and late-onset hypertensive disorders of pregnancy

**DOI:** 10.1038/s41598-025-02965-1

**Published:** 2025-05-24

**Authors:** Sho Tano, Tomomi Kotani, Tatsuo Inamura, Fumie Kinoshita, Kazuya Fuma, Seiko Matsuo, Masato Yoshihara, Kenji Imai, Shigeru Yoshida, Mamoru Yamashita, Yasuyuki Kishigami, Hidenori Oguchi, Hiroaki Kajiyama, Takafumi Ushida

**Affiliations:** 1 Department of Obstetrics and Gynecology, Nagoya University Graduate School of Medicine, Showa-ku Tsurumai 65, Nagoya, Aichi 466-8560 Japan; 2 Department of Obstetrics, Perinatal Medical Center, TOYOTA Memorial Hospital, Toyota, Aichi Japan; 3 Department of Obstetrics and Gynecology, Hamamatsu University School of Medicine, Hamamatsu, Shizuoka Japan; 4 Data Science Division, Data Coordinating Center, Department of Advanced Medicine, Nagoya University Hospital, Nagoya, Aichi Japan; 5 Kishokai Medical Corporation, Nagoya, Aichi Japan

**Keywords:** Overweight, Obesity, Glucose fluctuation, Preeclampsia, Preventive medicine, Endocrinology, Cardiovascular biology, Translational research

## Abstract

To evaluate the role of high glucose variability (High-GV) in early pregnancy as a potential mediating factor between pre-pregnancy overweight/obesity and late-onset HDP (LoHDP), where effective preventive strategies remain limited. This multicenter retrospective study analyzed data from 802 pregnancies across 14 facilities. Pregnancies with a 75 g-OGTT performed by 20 weeks of gestation were included. Structural equation modeling (SEM) was used to evaluate direct and indirect effects of body mass index (BMI), High-GV, and covariates (e.g., age, ART, primiparity) on LoHDP. Overweight/obese women had significantly higher rates of High-GV (26.1 vs. 16.4%, *p* = 0.001) and LoHDP (17.6 vs. 7.9%, *p* < 0.001) compared to non-overweight/obese women. SEM revealed that BMI influenced LoHDP through both direct and indirect pathways. BMI had a direct effect on LoHDP (β = 0.20, *p* < 0.01), and an indirect effect mediated by High-GV, with BMI significantly associated with High-GV (β = 0.15, *p* < 0.01), and High-GV positively associated with LoHDP (β = 0.12, *p* < 0.01). In Non-GDM pregnancies, High-GV showed an even stronger association with LoHDP (β = 0.25, *p* < 0.001). This study identifies High-GV as a key mediator linking pre-pregnancy overweight/obesity to LoHDP. These findings suggest that targeting glucose variability in early pregnancy could mitigate LoHDP risk, particularly in overweight/obese women, regardless of GDM status. Future preventive strategies should integrate multifaceted approaches addressing maternal BMI and glucose regulation to improve maternal and neonatal outcomes.

## Introduction

Hypertensive disorders of pregnancy (HDP) include conditions in which hypertension develops after 20 weeks of gestation, with pre-pregnancy overweight and obesity (overweight/obesity) being remarkable risk factors^1^. Recent studies have increasingly highlighted distinct pathophysiological differences between early-onset HDP (EoHDP), occurring before 34 weeks of gestation, and late-onset HDP (LoHDP), occurring after 34 weeks^2–4^. Overweight/obesity is more strongly associated with LoHDP, the more frequent subtype, than with EoHDP^2,5–10^. Accordingly, addressing pre-pregnancy overweight/obesity is expected to reduce the risk of HDP, and particularly of LoHDP.

Currently, low-dose aspirin is the primary preventive strategy available during pregnancy and is mainly targeted at preventing EoHDP, the more severe subtype^11–13^. Conversely, for LoHDP, the preventive approach during pregnancy is to reduce gestational weight gain (GWG)^14–16^, although evidence supporting specific methods to achieve appropriate GWG is lacking^16–18^. LoHDP has a similar long-term impact on women’s health postpartum than EoHDP^19^. Accordingly, understanding the mechanism by which overweight/obesity contributes to LoHDP onset is crucial for developing comprehensive preventive measures available during pregnancy.

The present study focused on high glucose variability (High-GV), which is increasingly being recognized as a risk factor for cardiovascular diseases (CVD)^20,21^, mainly due to its link with endothelial dysfunction, a mechanism shared with HDP^22–24^. Our previous report concluded that High-GV in early pregnancy was independently associated with subsequent LoHDP development^25^. However, we had not yet clarified what contributes to the High-GV in early pregnancy.

Research on glucose metabolism has identified overweight/obesity as a significant contributor to increased High-GV^26,27^. Furthermore, High-GV has been established as an intermediate factor between overweight/obesity and CVD^21,28,29^. Therefore, this study aims to analyze whether High-GV could serve as a mediating factor in the relationship between prepregnant overweight/obesity and LoHDP in non-diabetic pregnant women.

## Results

### Baseline characteristics and outcomes

Table 1 presents the baseline characteristics and pregnancy outcomes of the overweight/obesity (n = 272) and non-overweight/obesity (n = 530) groups. The mean maternal age was similar between the two groups (33.3 ± 4.9 years vs. 32.6 ± 5.2 years, *p* = 0.073). The rate of primiparity was lower in overweight/obesity group (38.6 vs. 47.2%, *p* < 0.001). The rates of assisted reproductive technology (ART) use was similar between the groups, with no significant difference (11.4% vs. 14.6%, *p* = 0.215). However, a higher percentage of women in the overweight/obesity group were classified as High-GV and GDM (26.1 vs. 16.4%, *p* = 0.001 for High-GV; and 61.8 vs. 50.0%, *p* = 0.002 for GDM). Regarding HDP, the incidence of LoHDP was significantly higher in the overweight/obesity group compared to the Non- overweight/obesity group (17.6 vs. 7.9%, *p* < 0.001).

**Table 1:** Baseline characteristics and outcomes.

	Overweight/obesity (n = 272)	Non-overweight/obesity (n = 530)	*p*-value
Maternal age, years	33.3 ± 4.9	32.6 ± 5.2	0.073
**Pre-regnant BMI, kg/m**^**2**^	**27.1 ± 4.0**	**20.1 ± 1.6**	**–**
Primiparity	**105 (38.6)**	**250 (47.2)**	0.021*
ART	31 (11.4)	77 (14.6)	0.215
GA at OGTT, weeks	13.5 ± 1.8	13.5 ± 1.7	0.899
**FPG, mg/dL**	**91.9 ± 9.8**	**87.8 ± 9.9**	< 0.001*
**1hPG, mg/dL**	**155.8 ± 30.2**	**144.8 ± 32.2**	< 0.001*
**2hPG, mg/dL**	**134.4 ± 25.3**	**129.7 ± 28.1**	0.008*
**Initial-increase, mg/dL**	**63.9 ± 28.8**	**57.0 ± 32.3**	0.001*
**Subsequent-decrease, mg/dL**	**21.4 ± 25.0**	**15.1 ± 27.2**	< 0.001*
**High-GV**	**71 (26.1)**	**87 (16.4)**	0.001*
**GDM**	**168 (61.8)**	**265 (50.0)**	0.002*
**HDP**	**65 (23.9)**	**50 (9.4)**	< 0.001*
**LoHDP**	**48 (17.6)**	**42 (7.9)**	< 0.001*
**EoHDP**	**17 (6.3)**	**8 (1.5)**	
GA at delivery, wks	39.3 ± 2.0	39.4 ± 1.6	0.233
**Birth weight, g**	**3,142 ± 504**	**3,054 ± 431**	0.014*

Continuous variables were represented as mean ± standard division. Categorical variables were represented as n (%).

*High-GV* high glucose variability, *BMI* body mass index, *ART* assisted reproductive technology, *GA* gestational age, *OGTT* oral glucose tolerance test, *FPG* fasting plasma glucose, *1hPG* 1-h post-load plasma glucose, *2hPG* 2 h post-load plasma glucose, *GV* glucose variability, *GDM* gestational diabetes mellitus, *HDP* hypertensive disorders of pregnancy, *EoHDP* early-onset HDP, *LoHDP* late-onset HDP.

The statistically significant results are indicated in bold, with asterisks (*) accompanying the corresponding *p*-values.

### Association between overweight and glucose parameters

**Fig. 1:**
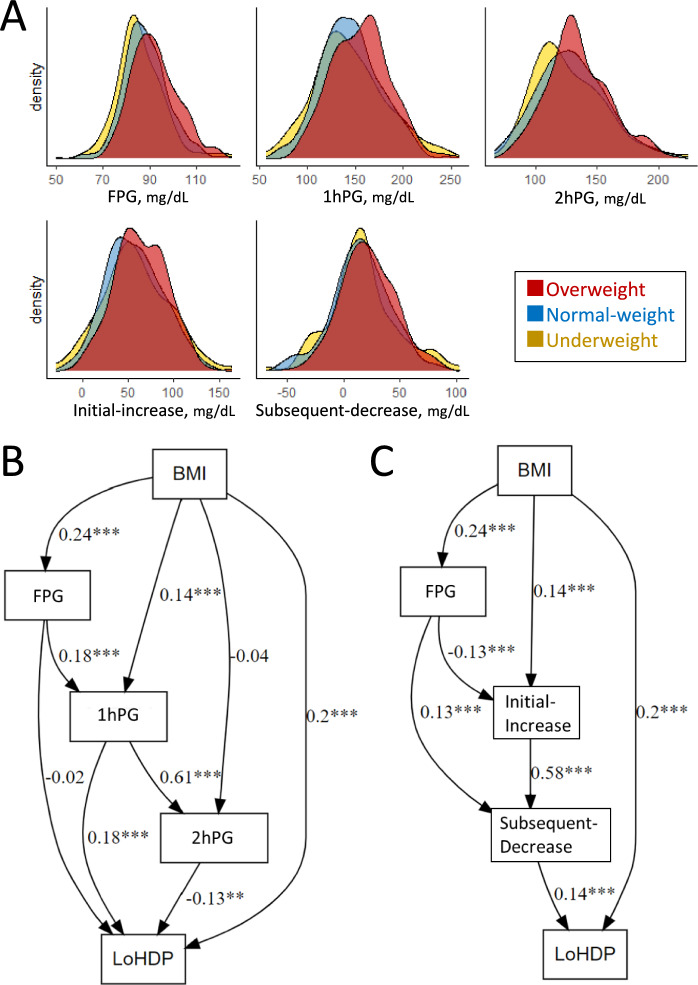
Relationships between OGTT parameters, glucose variability, and late-onset hypertensive disorders of pregnancy. (**A**), The X-axis of the density plot represents the value of 75 g-OGTT parameters. Red, blue, and yellow parts represent the overweight, normal-weight, and underweight, respectively. (**B**,**C**) Each panel visually represents the hypothesized relationships in the SEMs. Model 1 presents the SEM results exploring the relationships between OGTT glucose parameters (FPG, 1hPG, 2hPG) and LoHDP, with BMI as an influencing factor (**B**). Model 2 the relationship between BMI and LoHDP from the perspective of GV (**C**). The standardized regression coefficients (β) indicate the strength and direction of the relationships, while the asterisks highlight the statistical significance of these relationships. Significance levels are denoted by asterisks: *p < 0.05, **p < 0.01, ***p < 0.001. *BMI* body mass index, *FPG* fasting plasma glucose, *1hPG* 1-h post-load plasma glucose, *2hPG* 2-h post-load plasma glucose, *LoHDP* late-onset hypertensive disorders of pregnancy.

The distributions of 75 g-OGTT parameters were compared among overweight (red), normal-weight (blue), and underweignt (yellow) groups using the density plot (Fig. 1A). Overweight/obesity individuals demonstrated higher levels of fasting plasma glucose level (FPG), 1 h post-load plasma glucose (1hPG), and 2 h post-load plasma glucose (2hPG) compared to non-overweight/obesity groups. The magnitude of the initial-increase was also more pronounced in overweight/obesity participants, indicating a steeper glucose response following the oral glucose load. However, the subsequent-decrease did not show notable differences.

### Structural equation model

Model 1 explored the relationships between OGTT glucose parameters (FPG, 1hPG, 2hPG) and LoHDP, with body mass index (BMI) as an influencing factor using structural equation model (SEM) analyses (Fig. 1B and Table S1). BMI exhibited a significant direct effect on FPG (β = 0.24, *p* < 0.001) and 1hPG (β = 0.14, *p* < 0.001). Positive relationships were observed between FPG, 1hPG, and 2hPG. Interestingly, FPG did not show a significant direct effect on LoHDP, while 1hPG had a positive association with LoHDP (β = 0.18, *p* < 0.01), and 2hPG exhibited a negative association (β = -0.13, *p* = 0.003). Additionally, BMI had a significant direct effect on LoHDP (β = 0.20, *p* < 0.001), independent of glucose parameters. Model 2 re-evaluates the relationship between BMI and LoHDP from the perspective of GV (Fig. 1C and Table S1). The SEM results align with and support the findings from Model 1. BMI showed a significant direct effect on initial-increase (β = 0.14, *p* < 0.001), which in turn strongly predicted subsequent-decrease (β = 0.58, *p* < 0.001). Subsequent-decrease also had a significant positive association with LoHDP (β = 0.14, *p* < 0.001). These results indicate that BMI influences LoHDP indirectly through its effects on initial-increase and subsequent-decrease, in addition to its direct effect on LoHDP (β = 0.20, *p* < 0.001).

**Fig. 2:**
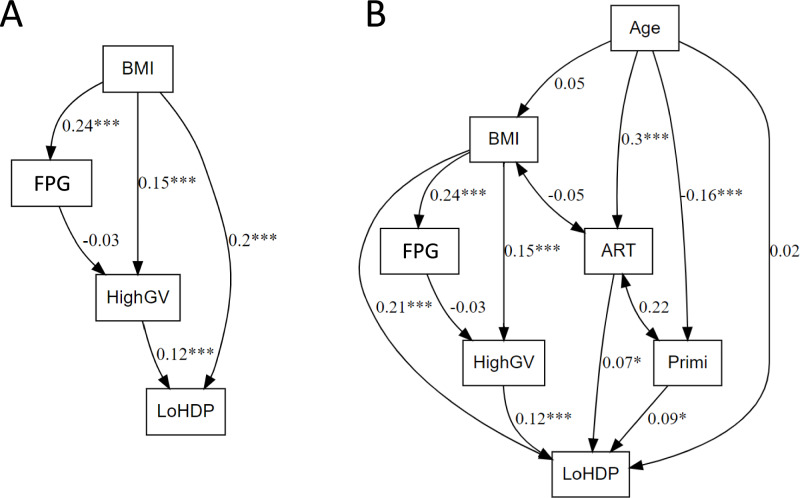
Direct and indirect effects of BMI and glucose variability on late-onset hypertensive disorders of pregnancy. Each panel visually represents the hypothesized relationships in the SEMs. Model 3 illustrating the relationships between BMI,FPG, High-GV, and LoHDP (**A**). Model 4 includes Age, ART, and Primi into the Model 3 (**B**). The standardized regression coefficients (β) indicate the strength and direction of the relationships, while the asterisks highlight the statistical significance of these relationships. Significance levels are denoted by asterisks: *p < 0.05, **p < 0.01, ***p < 0.001. *BMI* body mass index, *FPG* fasting plasma glucose, *High-GV* high glycemic variability, *LoHDP* late-onset hypertensive disorders of pregnancy, *ART* assisted reproductive technology, *Primi* primiparity.

Model 3 evaluates the relationship between BMI and LoHDP by conceptualizing HighGV, defined as the condition where both the initial-increase and subsequent-decrease are maximized (Fig. 2A and Table S2). The SEM results indicate that BMI influences LoHDP through both direct and indirect pathways. BMI had a direct effect on LoHDP (β = 0.20, *p* < 0.001) and an indirect effect mediated by High-GV, where BMI influenced High-GV (β = 0.15, *p* < 0.001), and High-GV was positively associated with LoHDP (β = 0.12, *p* < 0.001). Model 4 expands on the findings from Model 3 by incorporating additional covariates, including age, ART, and primiparity (Fig. 2B and Table S2). The SEM results confirm that the relationships identified in Model 3 remain consistent even after adjusting for these factors. BMI continues to influence LoHDP through both direct and indirect pathways. BMI retains a direct effect on LoHDP (β = 0.21, *p* < 0.001) and an indirect effect mediated by High-GV, with a significant association between BMI and High-GV (β = 0.15, *p* < 0.001) and between High-GV and LoHDP (β = 0.12, *p* < 0.001).

Table S3 presents the fit indices for each model. The fit indices indicate that every model provided a good fit to the data (χ^2^/df ≤ 4.50, RMSEA ≤ 0.07, NFI ≥ 0.95, IFI ≥ 0.97, TLI ≥ 0.93, CFI ≥ 0.97, GFI = 0.99, AGFI ≥ 0.96, and SRMR ≤ 0.03), further supporting the robustness of the proposed SEM framework. These results underscore the importance of considering multiple components when analyzing factors influencing HDP, with High-GV emerging as a significant mediator between BMI and LoHDP.

### Subgroup-analysis

Table 2 summarizes the results of the subgroup analysis stratified by GDM status. In the Non-GDM subgroup, the frequency of High-GV was significantly higher in the overweight/obesity group compared to the non-overweight/obesity group. Specifically, 25 out of 104 participants in the overweight/obesity group (24.0%) had High-GV, while in the non-overweight/obesity group, the frequency was 25 out of 265 (9.4%). Additionally, among non-overweight/obesity individuals, 20.0% of participants with High-GV developed LoHDP, compared to 6.3% of those without High-GV (*p* = 0.013). Similarly, in the overweight/obesity group, 44.0% of participants with High-GV developed LoHDP, compared to 8.9% of those without High-GV (*p* < 0.001). In the GDM subgroup, differences were observed based on overweight/obesity status. Among non-overweight/obesity individuals, participants with High-GV had a higher frequency of LoHDP (16.1% [10/62]) compared to those without High-GV (5.9% [12/203]; *p* = 0.011). In contrast, among overweight/obesity individuals with GDM, no significant difference in LoHDP frequency was observed between those with High-GV (15.2% [7/46]) and those without High-GV (21.7% [10/46]; *p* = 0.583).

**Table 2:** Association between GDM, Overweight, High-GV, and HDP.

GDM	Overweight/obesity	High-GV	*N*	HDP *n* (%)	LoHDP *n* (%)	*p*-value for	
						HDP	LoHDP
−	−	−	240	**18 (7.5)**	**15 (6.3)**	0.035*	0.013*
		+	25	**5 (20.0)**	**5 (20.0)**		
	+	−	79	**11 (13.9)**	**7 (8.9)**	< 0.001*	< 0.001*
		+	25	**13 (52.0)**	**11 (44.0)**		
+	−	−	203	**16 (7.9)**	**12 (5.9)**	0.025*	0.011*
		+	62	**11 (17.7)**	**10 (16.1)**		
	+	−	122	31 (25.4)	23 (18.9)	0.621	0.583
		+	46	10 (21.7)	7 (15.2)		

*GDM* gestational diabetes mellitus, *GV* glucose variability, *HDP* hypertensive disorders of pregnancy, *LoHDP* late-onset HDP.

*N*: total number of participants in each category; *n*: number of events in each category. *p*-values are derived from χ^2^ tests conducted for each category.

The statistically significant results are indicated in bold, with asterisks (*) accompanying the corresponding *p*-values.

Figures S1 present the SEM analyses focusing on Non-GDM individuals, evaluating the relationships among BMI, HighGV, and LoHDP as in the analyses conducted for Fig. 2. These results indicate that the findings observed in Fig. 2 remain consistent in the Non-GDM subgroup. BMI retains a significant direct effect on HighGV (β = 0.20, *p* < 0.01), and HighGV continues to exhibit a strong positive association with LoHDP (β = 0.25, *p* < 0.001), demonstrating an even stronger trend compared to the broader cohort analyzed in Fig. 2.

## Discussion

This study contributes to the existing body of knowledge regarding the relationship between maternal characteristics and HDP. This is the first study to evaluate High-GV in early pregnancy as a mediating factor between pre-pregnancy overweight/obesity and development of LoHDP. By identifying High-GV as a critical intermediary, this study provides novel insights into mechanisms linking maternal pre-pregnancy BMI with HDP, and particularly LoHDP. Our findings underscore the importance of timely glucose regulation in pregnant women with higher BMI to mitigate the risk of LoHDP.

This study highlighted the complex interplay between the maternal BMI, High-GV, LoHDP, Age, ART, and primiparity. The inclusion of each additional variable provides a more comprehensive understanding of these relationships, with BMI consistently showing a strong influence on High-GV and LoHDP across all models. These results are consistent with those of previous research^8–10^. In non-pregnant individuals, high GV has been shown to contribute to the development of CVD^30^. Given that both CDV and HDP share vascular endothelial dysfunction as a central pathological feature^31^, it is plausible that high GV during pregnancy is also associated with the onset of HDP. Additionally, we demonstrated an association between High-GV and LoHDP even in non-GDM individuals. Nevertheless, GDM is reported to be associated with an increased risk of HDP^32,33^, and this may be explained by the higher prevalence of GDM observed in women with High-GV in this study. However, an interesting observation in our study was the lack of association between High-GV and LoHDP among overweight/obese women with GDM. This may be explained by distinct vascular and metabolic adaptations in this subgroup compared to non-overweight/obese women with GDM^34^. The coexistence of GDM with overweight/obesity leads to pre-existing vascular remodeling, potentially diminishing endothelial sensitivity to GV^34^. Consequently, the incidence of LoHDP remains consistent regardless of GV levels, suggesting that chronic endothelial dysfunction in overweight/obese women with GDM may blunt the vascular impact of GV.

LoHDP is also associated with lipid metabolism^35,36^. Although our study did not include lipid metabolism parameters, BMI and LoHDP had significant direct effects independent of High-GV, suggesting that factors related to lipid metabolism may be involved. Furthermore, the association between primiparity or ART and LoHDP was consistent with that of previous studies^1,37,38^.

Althogh women diagnosed with GDM before 20 weeks of pregnancy have poorer perinatal outcomes^39^, the evidence on how to evaluate and manage GDM when diagnosed at this stage is still limited, which is why OGTT is not widely recommended in early pregnancy^40^. However, from the perspective of LoHDP prevention, our study suggests that assessing and addressing GV evaluation could be a beneficial strategy. Previously, the mediating factors linking overweight/obesity and HDP remained unclear, limiting preventive strategies for HDP in overweight/obese pregnant women to weight management before or during pregnancy^41–44^. However, our study identified GV as a potential mediator, suggesting that a multifaceted, seamless preventive approach targeting glycemic control from the preconception period through pregnancy may now be explored.

This study has several strengths. First, the evaluation of a large and diverse dataset from multiple centers enhances the generalizability of the findings. The inclusion of data from both tertiary centers and private maternity facilities ensures a comprehensive representation of the population, accounting for various socioeconomic and healthcare backgrounds. Moreover, the use of SEM allowed for a nuanced exploration of the complex interrelationships among maternal characteristics and pregnancy outcomes^45^. Lastly, the rigorous exclusion criteria ensured that the study focused specifically on pregnant women without diabetes mellitus (DM), eliminating potential confounding effects of pre-existing DM and chronic hypertension. This targeted approach strengthens the validity of the conclusions drawn regarding the impact of BMI and High-GV on HDP.

### Limitations

Although this study offers valuable insights, several limitations should be considered when interpreting the results. First, the retrospective design may have introduced selection bias and analytical methods were applied, the bias inferent to retrospective studies cannot be completely ruled out. Second, relying on self-reported maternal height and weight to calculate pre-pregnancy BMI could introduce measurement errors. However, participants were weighed in the first trimester; therefore, discrepancy between self-reported and actual values was likely to be minimal. Third, the study population included only women with random blood glucose (RBG) ≥ 100 mg/dL in Japan, which may limit the generalizability of the findings to other populations or ethnic groups. The definition and clinical significance of High-GV may vary across populations due to racial and ethnic differences in glucose metabolism and pregnancy outcomes. Despite these limitations, the study makes an important contribution to improve our understanding of the relationship between maternal BMI, GV, and HDP, and highlights areas for further research and potential intervention.

## Conclusion

This study advances our understanding of the pathophysiological mechanisms linking maternal overweight/obesity to HDP, particularly LoHDP, through the intermediary role of High-GV. These findings provide a basis for more targeted and efficient preventive strategies, ultimately contributing to better maternal and neonatal health outcomes through the development of seamless and multifaceted preventive measures from pre- to post-conception. However, as the study included only women with RBG ≥ 100 mg/dL, these conclusions should be interpreted cautiously and validated in broader populations.

## Methods

### Study design and participants

**Fig. 3:**
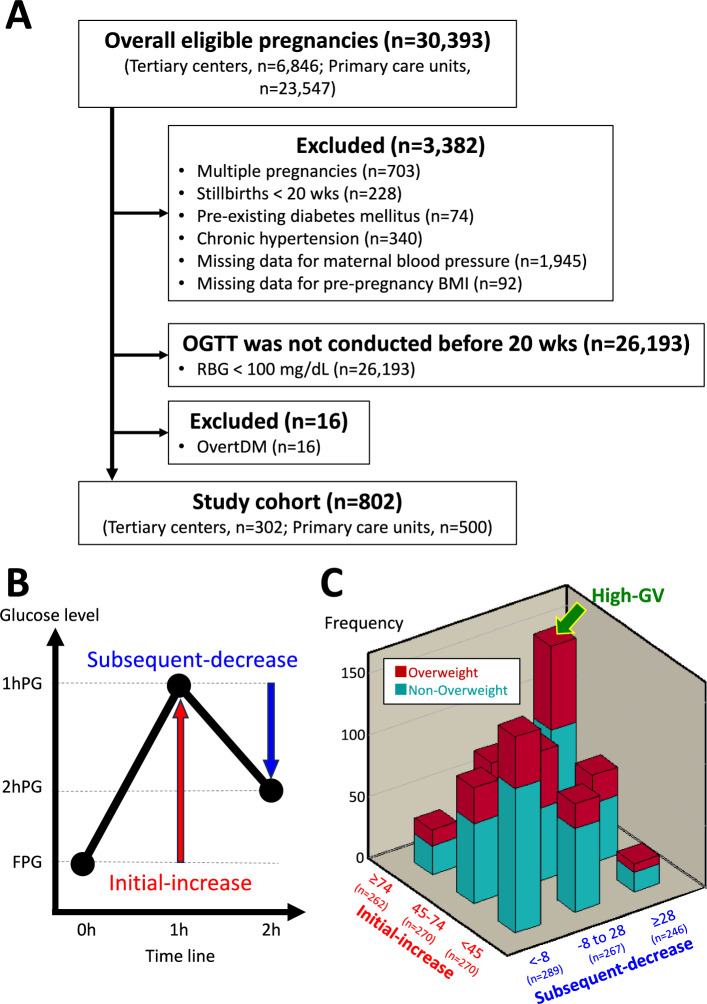
Characteristics of study participants. (**A**) Flowchart of study enrolment. Among 30,393 individuals, 802 were eligible after excluding 29,591 individuals. (**B**) Schematic representation of OGTT results and definition of initial-increase and subsequent-decrease. Initial-increase was defined as the increase in in blood glucose levels from FPG to 1hPG. Subsequent-decrease was defined as the decrease in blood glucose levels from 1 to 2hPG. (**C**) Three-dimensional bar chart representing the frequency of overweight and non-overweight (n = 802). The frequencies of overweight (red-cuboid) and non-overweight (blue-cuboid) are represented on the Z axis, with the classified initial-increase and subsequent-decrease forming the axes of X and Y, respectively. High-GV was defined as individuals with a significant initial-increase of ≥ 74 mg/dL with a notable subsequent-decrease of ≥ 28 mg/dL. *BMI* body mass index, *DM* diabetes mellitus, *RBG* random blood glucose level, *wk* weeks of gestation, *OGTT* oral glucose tolerance test, *FPG* fasting plasma glucose, *1hPG* 1-h post-load plasma glucose, *2hPG* 2-h post-load plasma glucose, *High-GV* high glycemic variability.

This multicenter retrospective study used electronic data from two tertiary centers (Nagoya University Hospital and TOYOTA Memorial Hospital, Aichi, Japan) and 12 private maternity facilities (Kishokai Medical Corporation, Aichi, Japan). The dataset included women aged ≥ 15 years who gave birth between 2009 and 2019, with available data from before 20 weeks of gestation. The study specifically included pregnancies where an 75 g-OGTT was performed by 20 weeks. The exclusion criteria were women with multiple pregnancies, stillbirth before 20 weeks, chronic hypertension, pre-existing DM, overt-DM, and missing data on blood pressure before 20 weeks and pre-pregnant BMI. Despite chronic hypertension being a subtype of HDP, this group was excluded to specifically assess the association between GV and the development of HDP (Fig. 3A).

This study was approved by the ethics board of Nagoya University Hospital (approval number: 2015–0415) following the guidelines of the Declaration of Helsinki and was conducted according to the STROBE guidelines. The requirement for informed consent was waived because of the retrospective study design.

### Definitions

HDP was defined as the development of new hypertension (systolic blood pressure of ≥ 140 mmHg or diastolic blood pressure of ≥ 90 mmHg) after 20 weeks of gestation^1,46^. HDP was classified as LoHDP if it occurred at ≥ 34 weeks, and EoHDP if it occurred at < 34 weeks^47,48^. GV was evaluated using initial-increase and subsequent-decrease (Fig. 3B). The initial-increase was defined as the increase in blood glucose levels from FPG to the 1hPG, and the “subsequent-decrease” was defined as the decrease from 1hPG to 2hPG^25,49^. High-GV was defined as initial-increas of ≥ 74 mg/dL (4.1 mmol/L) followed by a notable subsequent-decrease of ≥ 28 mg/dL (1.6 mmol/L), as shown in (Fig. 3C)^25,49^. These cutoff values were determined based on the tertiles of each respective change.

Self-reported maternal height and body weight were used to calculate pre-pregnant BMI (kg/m^2^). In this study, we defined individuals with a BMI of < 18.5 kg/m^2^ as underweight, and of ≥ 23.0 kg/m^2^ as overweight/obesity, in accordance with the World Health Organization criteria for the Asian population^50^. ART included in vitro fertilization or intracytoplasmic sperm injection.

### Diagnosis of overt-DM/GDM

Pregnant women were screened for RBG levels at approxymately 12–14 weeks of gestation, according to the guidelines; the cut-off value was set at RBG of ≥ 100 mg/dL (5.6 mmol/L)^17,51^. For screening-positive patients, the FPG level was measured usually within 1–2 weeks, and patients with FPG of ≥ 126 mg/dL (7.0 mmol/L) were diagnosed as overt-DM (excluded from this analysis). For non-overt-DM individuals, the 75 g-OGTT was conducted with cutoff values of ≥ 92 mg/dL (5.1 mmol/L) for FPG, ≥ 180 mg/dL (10 mmol/L) for 1hPG, and ≥ 153 mg/dL (8.5 mmol/L) for 2hPG^17^. GDM was diagnosed if at least one of the three aforementioned glycemic levels was above the threshold value^17^. In Japan, oral diabetes medications are contraindicated for pregnant women, so treatment was by diet and insulin injections.

### Outcome

The primary outcome is to determine whether GV in early pregnancy serves as a mediating factor in the relationship between pre-pregnancy BMI and the development of LoHDP in non-diabetic pregnant women.

### Statistical analysis

Baseline characteristics of overweight/obese and non-overweight/obese pregnant women were compared using the independent samples t-test and Chi-square test. Based on the clinical relevance, an initial SEM was constructed to synthesize path-reflective relationships between variables and to explore the direct and indirect effects of overweight status and GV on LoHDP. The analysis included the calculation of standardized beta coefficients (β) to quantify the strength and direction of these relationships, thereby providing a comprehensive understanding of both direct and mediated pathways within the model. The following ideal-fit-indices were used to evaluate the model: χ^2^/df < 3 [acceptable < 5], a root mean square error of approximation (RMSEA) < 0.05 [acceptable ≤ 0.08], normed fit index (NFI) ≥ 0.95 [acceptable ≥ 0.90], incremental fit index (IFI) ≥ 0.95 [acceptable ≥ 0.90], Tucker–Lewis index (TLI) ≥ 0.95 [acceptable ≥ 0.90], comparative fit index (CFI) ≥ 0.95 [acceptable ≥ 0.90], goodness of fit index (GFI) ≥ 0.95 [acceptable ≥ 0.90], adjusted goodness-of-fit index (AGFI) ≥ 0.90 [acceptable ≥ 0.85], and standardized root mean square residual (SRMR) < 0.05 [acceptable ≤ 0.08]^52–56^.

## Supplementary Information

Supplementary Information 1.

Supplementary Information 2.

Supplementary Information 3.

Supplementary Information 4.

## Data Availability

The raw data underlying the conclusions of this article can be accessed upon reasonable request, subject to the approval of Nagoya University, TOYOTA Memorial Hospital, and Kishokai Medical Corporation. Contact information: Sho Tano, tano.sho.v4@f.mail.nagoya-u.ac.jp.
