# Supplementary material for: Glucose variability as a key mediator in the relationship between pre-pregnancy overweight/obesity and late-onset hypertensive disorders of pregnancy
Source: Sci Rep. 2025 May 24;15:18123. doi: 10.1038/s41598-025-02965-1 (PMC12103585; doi:10.1038/s41598-025-02965-1)
Supplement: Supplementary file 1 — Supplementary Information 1. [file 41598_2025_2965_MOESM1_ESM.docx]

**Figure S1. Direct and Indirect Effects of BMI and Glucose Variability on Late-Onset Hypertensive Disorders of Pregnancy among Non-GDM Individuals**


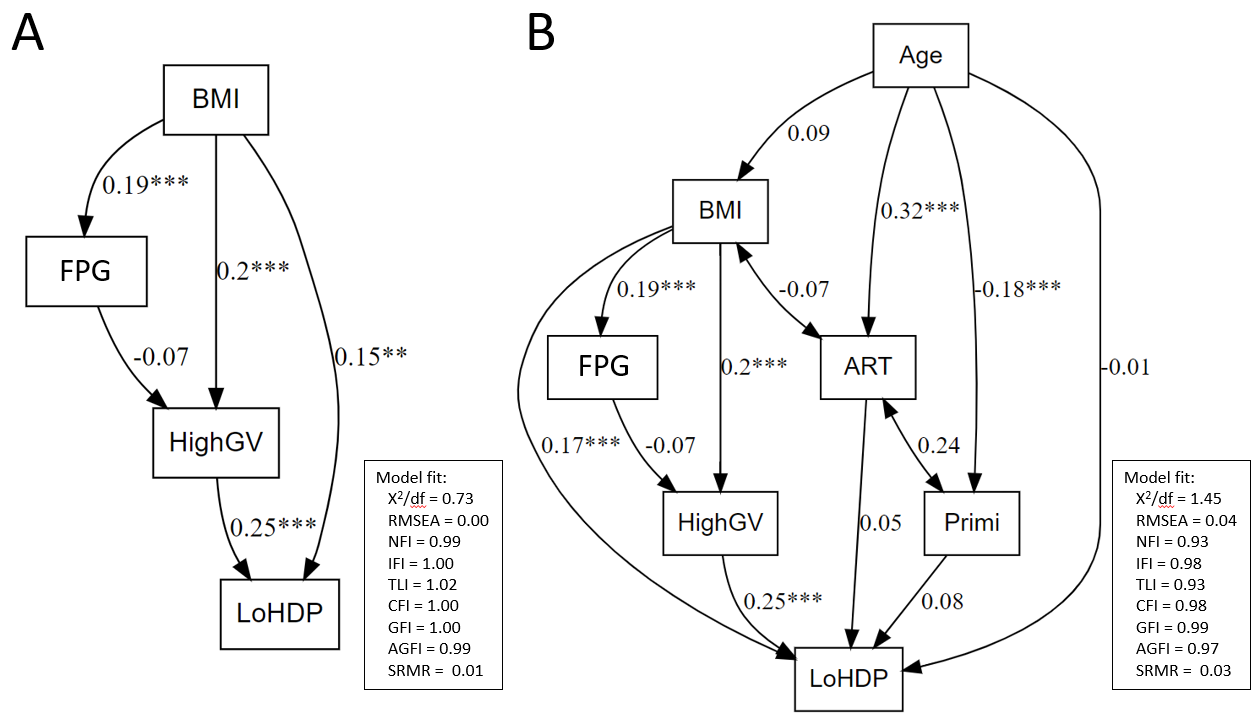


Each panel visually represents the hypothesized relationships in the SEMs. Model 3 illustrating the relationships between BMI; FPG, High-GV, and LoHDP (A). Model 4 includes Age, ART, and Primi into the Model 3 (B). The standardized regression coefficients (β) indicate the strength and direction of the relationships, while the asterisks highlight the statistical significance of these relationships. Significance levels are denoted by asterisks: **p* < 0.05, ***p* < 0.01, ****p* <0.001.

BMI, body mass index; FPG, fasting plasma glucose; High-GV, high glycemic variability; LoHDP, late-onset hypertensive disorders of pregnancy; ART, assisted reproductive technology; Primi, primiparity.
