# Supplementary material for: Glucose variability as a key mediator in the relationship between pre-pregnancy overweight/obesity and late-onset hypertensive disorders of pregnancy
Source: Sci Rep. 2025 May 24;15:18123. doi: 10.1038/s41598-025-02965-1 (PMC12103585; doi:10.1038/s41598-025-02965-1)
Supplement: Supplementary file 2 — Supplementary Information 2. [file 41598_2025_2965_MOESM2_ESM.docx]

Table S1. Structural Equation Modeling Results

| Response |  | Predictor | Model 1 | | |  | Model 2 | | |
| --- | --- | --- | --- | --- | --- | --- | --- | --- | --- |
|  |  |  | β | SE | *p*-value |  | β | SE | *p*-value |
| FPG | ← | BMI | **0.24** | **0.08** | **<0.001*** |  | **0.24** | **0.08** | **<0.001*** |
| 1hPG | ← | BMI | **0.14** | **0.26** | **<0.001*** |  | - | - | - |
| 2hPG | ← | BMI | -0.04 | 0.18 | 0.123 |  | - | - | - |
| LoHDP | ← | BMI | **0.20** | **0.00** | **<0.001*** |  | **0.20** | **0.00** | **<0.001*** |
| 1hPG | ← | FPG | **0.18** | **0.11** | **<0.001*** |  | - | - | - |
| LoHDP | ← | FPG | -0.02 | 0.00 | 0.513 |  | - | - | - |
| 2hPG | ← | 1hPG | 0.61 | 0.02 | **<0.001*** |  | - | - | - |
| LoHDP | ← | 1hPG | 0.18 | 0.00 | **<0.001*** |  | - | - | - |
| LoHDP | ← | 2hPG | **-0.13** | **0.00** | **0.003*** |  | - | - | - |
| Initial-increase | ← | BMI | - | - | - |  | **0.14** | **0.26** | **<0.001*** |
| Initial-increase | ← | FPG |  |  |  |  | **-0.13** | **0.11** | **<0.001*** |
| Subsequent-decrease | ← | FPG |  |  |  |  | **0.13** | **0.08** | **<0.001*** |
| Subsequent-decrease | ← | Initial-increase | - | - | - |  | **0.58** | **0.03** | **<0.001*** |
| LoHDP | ← | Subsequent-decrease | - | - | - |  | **0.14** | **0.00** | **<0.001*** |

GV, glucose variability; BMI, body mass index; Primi, primiparity; ART, assisted reproductive technology; LoHDP, late-onset hypertensive disorders of pregnancy; SE, standardized error.
