# Supplementary material for: Glucose variability as a key mediator in the relationship between pre-pregnancy overweight/obesity and late-onset hypertensive disorders of pregnancy
Source: Sci Rep. 2025 May 24;15:18123. doi: 10.1038/s41598-025-02965-1 (PMC12103585; doi:10.1038/s41598-025-02965-1)
Supplement: Supplementary file 3 — Supplementary Information 3. [file 41598_2025_2965_MOESM3_ESM.docx]

Table S2. Structural Equation Modeling Results

| Response |  | Predictor | Model 3 | | |  | Model 4 | | |
| --- | --- | --- | --- | --- | --- | --- | --- | --- | --- |
|  |  |  | β | SE | *p*-value |  | β | SE | *p*-value |
| FPG | ← | BMI | **0.24** | **0.03** | **<0.001*** |  | **0.24** | **0.03** | **<0.001*** |
| HighGV | ← | BMI | **0.15** | **0.04** | **<0.001*** |  | **0.15** | **0.04** | **<0.001*** |
| LoHDP | ← | BMI | **0.20** | **0.03** | **<0.001*** |  | **0.21** | **0.03** | **<0.001*** |
| HighGV | ← | FPG | -0.03 | 0.04 | 0.358 |  | -0.03 | 0.04 | 0.396 |
| LoHDP | ← | HighGV | **0.12** | **0.03** | **<0.001*** |  | **0.12** | **0.03** | **<0.001*** |
| BMI | ← | Age | - | - | - |  | 0.05 | 0.04 | 0.187 |
| ART | ← | Age | - | - | - |  | **0.30** | **0.03** | **<0.001*** |
| Primi | ← | Age | - | - | - |  | **-0.16** | **0.03** | **<0.001*** |
| LoHDP | ← | Age | - | - | - |  | 0.02 | 0.04 | 0.604 |
| ART | ←→ | BMI | - | - | - |  | -0.05 | 0.03 | 0.124 |
| Primi | ←→ | ART | - | - | - |  | **0.22** | **0.03** | **<0.001*** |
| LoHDP | ← | ART | - | - | - |  | **0.07** | **0.04** | **0.042*** |
| Primi | ← | LoHDP | - | - | - |  | **0.09** | **0.04** | **0.012*** |

GV, glucose variability; BMI, body mass index; Primi, primiparity; ART, assisted reproductive technology; LoHDP, late-onset hypertensive disorders of pregnancy; SE, standardized error.
