# Supplementary material for: Glucose variability as a key mediator in the relationship between pre-pregnancy overweight/obesity and late-onset hypertensive disorders of pregnancy
Source: Sci Rep. 2025 May 24;15:18123. doi: 10.1038/s41598-025-02965-1 (PMC12103585; doi:10.1038/s41598-025-02965-1)
Supplement: Supplementary file 4 — Supplementary Information 4. [file 41598_2025_2965_MOESM4_ESM.docx]

Table S3. Fit Indices for SEM

|  | Ideal Fit Indices | Acceptable Fit Indices | Model 1 | Model 2 | Model 3 | Model 4 |
| --- | --- | --- | --- | --- | --- | --- |
| χ^2^/df | ≤3 | ≤5 | 4.50 | 1.53 | 0.05 | 1.93 |
| RMSEA | <0.05 | ≤0.08 | 0.07 | 0.03 | 0.00 | 0.03 |
| NFI | ≥0.95 | ≥0.90 | 0.99 | 0.99 | 1.00 | 0.95 |
| IFI | ≥0.95 | ≥0.90 | 0.99 | 1.00 | 1.01 | 0.97 |
| TLI | ≥0.95 | ≥0.90 | 0.93 | 0.99 | 1.05 | 0.93 |
| CFI | ≥0.95 | ≥0.90 | 0.99 | 1.00 | 1.00 | 0.97 |
| GFI | ≥0.95 | ≥0.90 | 1.00 | 1.00 | 1.00 | 0.99 |
| AGFI | ≥0.90 | ≥0.85 | 0.96 | 0.99 | 1.00 | 0.98 |
| SRMR | <0.05 | ≤0.08 | 0.02 | 0.02 | 0.00 | 0.03 |

RMSEA, Root Mean Square Error of Approximation; NFI, Normed Fit Index; IFI, Incremental Fit Index; TLI, Tucker–Lewis index; CFI, Comparative Fit Index; GFI, Goodness of Fit Index; AGFI, Adjusted Goodness-of-Fit Index; and SRMR, Standardized Root Mean Square Residual.
